# Research Note: A sip of stress. Effects of corticosterone supplementation in drinking water on feather corticosterone concentrations in layer pullets

**DOI:** 10.1016/j.psj.2021.101361

**Published:** 2021-06-30

**Authors:** T. Bartels, J. Berk, K. Cramer, E. Kanitz, W. Otten

**Affiliations:** ⁎ Friedrich-Loeffler-Institut, Institute of Animal Welfare and Animal Husbandry, Celle, Germany; † University of Leipzig, Clinic for Birds and Reptiles, Leipzig, Germany; ‡ Leibniz Institute for Farm Animal Biology (FBN), Institute of Behavioural Physiology, Dummerstorf, Germany

**Keywords:** laying hen, stress, glucocorticoid, feather corticosterone, animal welfare

## Abstract

The measurement of feather corticosterone concentrations (**fCORT**) is a comparatively new method for the evaluation of stress in wild and captive birds and may be a useful indicator in animal welfare research. The aim of this study was to assess the suitability of fCORT as an indicator of stress, and for this purpose a prolonged stress period was experimentally simulated by oral intake of corticosterone via drinking water and corticosterone concentrations were analyzed in feathers grown during this period. Layer pullets of both a control group (n = 20) and a CORT group (n = 20) were offered drinking water ad libitum throughout the entire experimental phase. The drinking water of the CORT group was supplemented with corticosterone at a concentration of 20 mg/l from the 64th to the 114th day of life. The vaned parts of the primaries 5 (**P5s**) were clipped on d 114 and fCORT was analyzed by ELISA after extraction. Body weights increased from day 64 until d 114 in both groups, however, at the end of the experiment, mean body mass in the CORT group was significantly lower than in the control group (*P* < 0.001). Pullets of the CORT group also showed shorter and lighter P5s as well as a retarded molt of the primaries. The supplementation of drinking water with corticosterone increased the average fCORT in the P5s of the CORT pullets compared with the control group (median: 110.3 pg/mm [interquartile range (IQR): 47.2] vs. 10.0 pg/mm [IQR: 2.5], *P* < 0.001). The results show that experimentally increased systemic corticosterone concentrations over a period of seven weeks in layer pullets are reflected in corticosterone concentrations of feathers grown during that time. This indicates that the measurement of fCORT may be a useful and minimally invasive tool for the evaluation of long-term stress in chicken and provides the basis for further investigations on its use in animal welfare research.

## INTRODUCTION

The exposure to stress is associated with a wide range of physiological responses and the key neuroendocrine axis in mediating these effects is the hypothalamic-pituitary-adrenal (**HPA**) axis. Its activation in birds leads to the species-specific release of the glucocorticoid (**GC**) hormone corticosterone. An important issue of the consequences of GC-mediated effects on health is the duration of the hormonal stress response. Whereas occasional, acute GC reactions are part of the organism's normal adaptation to stressful situations, cumulative stresses can be associated with a range of maladaptive responses due to frequent HPA axis activation or long-term changes or both in basal GC secretion. Thus, information on the long-term secretion of GCs and the deviation from reference ranges in blood and other sources are suitable biomarkers for physiological evidence of stress (Scanes, 2016).

The measurement of feather corticosterone concentration (**fCORT**) is a comparatively new and increasingly used method in stress research in wild and captive birds as well as in poultry (Carbajal et al., 2014; Romero and Fairhurst, 2016; Häffelin et al., 2020; Leishman et al., 2020; Monclús et al., 2020; Reese et al., 2020). Numerous studies have reported a stable incorporation of corticosterone in feathers and a positive correlation between corticosterone concentrations in feathers and in peripheral blood (Bortolotti et al., 2008; Romero and Fairhurst, 2016). Thus, fCORT could be a useful indicator of stress in animal welfare research. It provides the potential to retrospectively assess stress in birds over several weeks in one sample using a minimally invasive sampling procedure (Romero and Fairhurst, 2016).

However, there is a need for further evaluation of the suitability of fCORT as an indicator of long-term stress in poultry. To this end, supplementation of drinking water with corticosterone can be used to simulate stress-induced elevations of systemic corticosterone levels (Shini et al., 2009; Weimer et al., 2018). An increased incorporation of corticosterone into the growing feather should be detectable after prolonged oral intake of corticosterone via drinking water, and this model could be further used for the investigation of temporal dynamics of corticosterone incorporation and of different stress intensities. Therefore, it was the aim of this study to investigate the applicability of fCORT as an indicator of long-term stress.

For this purpose, a prolonged stress period was experimentally simulated by supplementing drinking water with corticosterone and corticosterone concentrations were analyzed in feathers grown during this period. The administration of corticosterone via drinking water is considerably less stressful for the pullets than a direct oral application, which involves compulsory handling measures and may therefore cause stress in the birds (Wein et al., 2017). In this model of unaffected corticosterone administration, the effects of other stressors are eliminated because no coercive measures (e.g., daily capture and restraint of the animal, forced opening of the beak for corticosterone application) are required. In addition, the corticosterone intake is distributed over the activity phase according to the birds’ drinking requirements.

## ANIMALS, MATERIALS, AND METHODS

All procedures performed in this study involving animal handling and treatments were in accordance with the German animal protection law and were approved by the local ethics committee (Lower Saxony State Office for Consumer Protection and Food Safety, Oldenburg, Germany; LAVES/AZ 33.19-42502-04-19/3092).

### Animals, Housing, and Management

Forty white-feathered layer pullets (Lohmann Selected Leghorn [**LSL**]) with intact beaks were purchased from a rearing farm at 8 wk of age and subsequently housed at the Institute for Animal Welfare and Animal Husbandry. None of the birds showed clinical signs of disease or physical impairment. The pullets were individually marked with wing bands and allocated to 2 barn compartments (5.4 m² each, 20 chicken per compartment), where they were kept until the end of the experiment. The husbandry conditions of the pullets were compliant with the German animal welfare legislation. Each compartment was strewn with wood shavings and equipped with perches. Compartments were climate-controlled and shielded lightproof from external light. Lighting was provided at an intensity of at least 20 lx and the lighting program was set to a 16L:8D light-dark cycle. The daily photoperiod lasted from 5:00 a.m. to 9:00 p.m. and included 20-min twilight phase in the mornings and evenings. The chickens were fed ad libitum with a home-made complete feed for layer pullets. Weight controls were performed on d 64 and 114.

### Experimental Design

The pullets of both the control (n = 20) and CORT group (n = 20) were offered drinking water ad libitum throughout the experimental period. To ensure an adequate drinking water supply, both compartments were equipped each with 2 nipple drinkers respectively (volume: 3,000 mL), each with 3 drinking nipples with drip cups. Starting from 64 d of life, the drinking water of pullets in the CORT group was supplemented with corticosterone (purity ≥92%, C2502, Sigma-Aldrich Chemie GmbH, Schnelldorf, Germany) at a concentration of 20 mg/l, as also applied by Shini et al. (2009) and Weimer et al. (2018). At this time, primaries 4 (**P4s**) of the first feather generation had already been partially shed and the renewed P4s were already partially recognizable as pin feathers. The experimental period lasted until d 114 of life. This time point was chosen because the P5s were then fully grown, recognizable by the dried dermal pulp inside the feather quill, and could be removed without tissue injury. Following Bortolotti (2010), only the vaned portion of the feather was used for further analyses. For each individual, the P5s of both wings were clipped within the range of the feather quill beneath the superior umbilicus using a wire cutter. Afterward, the feather vanes were macroscopically examined for structural alterations (e.g., fault bars) and stored protected from light until further processing.

### Feather Corticosterone Extraction and Measurement

Prior to analysis, all feathers were checked for integrity. To remove surface contaminants, intact feathers were washed by swirling and immersing in a dilute soap (1%) and ultrapure water solution for 30 s (Bortolotti et al., 2008; Jenni-Eiermann et al., 2015). Subsequently, feathers were rinsed twice for 30 s in ultrapure water and air-dried overnight.

Corticosterone extraction followed modified methods previously described by Bortolotti et al. (2008). From the right P5, the calamus was removed and the remaining feather length and weight were recorded. The feather was coarsely chopped with scissors, transferred into 10 mL stainless steel grinding jars with two 12-mm stainless steel grinding balls, frozen in liquid nitrogen, and pulverized using a Retsch ball mill (MM 400, Retsch GmbH, Haan, Germany) for 2 min at 30 Hz. Approximately 50 mg of feather powder was weighed out, transferred into 15 mL polypropylene tubes and mixed with 5 mL methanol (HPLC grade). The samples were placed in an ultrasonic water bath (Sonorex Digitec, Bandelin, Berlin, Germany) at room temperature for 30 min and then incubated in a slow-shaking 50°C water bath (Stuart SBS40, Barloworld Scientific, Staffordshire, UK) for 18 to 24 h. After extraction, samples were centrifuged at 5,000 rpm for 2 min, and 4 mL of each methanol extract was aliquoted into a 5 mL polypropylene tube and then vaporized using a vacuum evaporator (SC210A SpeedVac, Thermo Fisher Scientific, Waltham, MA).

For corticosterone analyses, the extract residues were reconstituted with 0.4 mL of the buffer solution of the assay kit used. Feather corticosterone concentrations were analyzed in duplicate using a species-specific ELISA kit for corticosterone quantification in chicken (CSB-E11991C, CUSABIO, Wuhan, China) according to the manufacturer's instructions. All measured values were related to both feather mass and feather length and corticosterone concentrations were presented in pg/mm.

To avoid the small sample artifacts (Lattin et al., 2011; Berk et al., 2016), the corticosterone concentration was previously tested as a function of feather mass (range 10–100 mg) indicating 50 mg as optimal. In addition, the methanol extraction efficiency of the feather mass used in this study was determined (1, 2, 5, 8, 10, and 12 mL methanol), with a methanol volume of 5 mL being optimal for sample handling and duration of evaporation. Accuracy was tested by comparing the standard curve (n = 5 standard concentrations) with a serial dilution of pooled samples (n = 4 concentrations) after log-transformation. The testing revealed linearity and parallelism of the standard and dilution curves, described by the formula y_standard_ = 0.97x + 0.10 with R^2^ = 0.995 and y_dilution_ = 0.96x + 0.15 with R^2^ = 0.972. Recovery was measured by spiking known amounts of corticosterone standards (n = 4 concentrations) to pooled samples and the average recovery was 0.84 ± 0.21. The manufacturer provided no information about the cross-reactivity of the antibody with other steroids. The detection limit of the procedure was 7 pg/mm based on the lowest standard of the assay kit. Intra- and interassay coefficients of variation of the analyzed samples were <10% and <15%, respectively.

### Statistical Analysis

All statistical analyses were performed using the software SPSS Statistics (version 25; IBM, Armonk, NY). Data distribution was tested for normality with the Shapiro-Wilk test. Data were checked for significant group differences using the *t* test for independent samples when normally distributed. Levene's test was applied to test for homogeneity of variances. For non-normally distributed data, Mann Whitney U test was performed. Development of body weight within the two study groups was analyzed by *t* test for paired samples. Values of *P* < 0.05 were considered significant.

## RESULTS AND DISCUSSION

Previous studies of Shini et al. (2008a,b) have shown that administration of corticosterone via drinking water increases circulating corticosterone above baseline levels and induces effects similar to those observed in responses to stressors. In the present study, weight gain in layer pullets was significantly decreased after oral application of corticosterone via drinking water. The initial mean body mass of pullets in the CORT group (672.4 g [± 45.3 g]) on d 64 was not significantly different from that of pullets of the control group (693.5 g [± 46.1 g]). However, at the end of the experiment on day 114, mean body mass was significantly lower (*P* < 0.001) in the CORT group (1,030.6 g [± 72.0 g]) than in the control group (1,178.8 g [±51.7 g]). These results correspond with findings from Shini et al. (2009), who also found significantly decreased body weights in chickens due to catabolic effects after long-term corticosterone administration.

**Figure 1:**
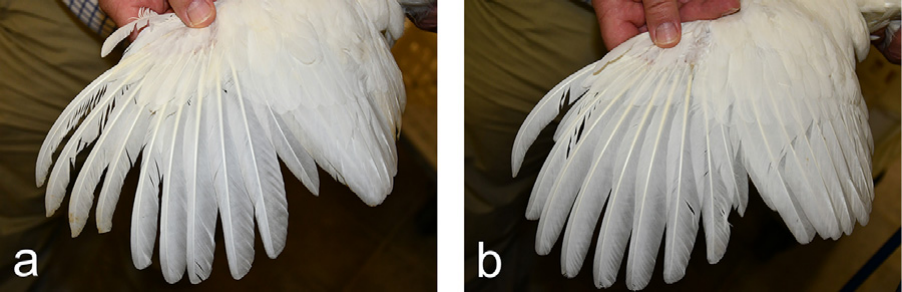
Molting status of LSL layer pullets of the CORT group (A) and the control group (B) at d 114. Dorsal view on the left wing. In pullets of the CORT group P1 to P5 of the second feather generation are fully-grown, P6 is shed and P7 to P10 still belong to the first feather generation. Pullets of the control group show seven mature primaries of the second feather generation. P8 is growing and P9 is shed. Only P10 belongs still to the first feather generation.

Significant differences between the CORT group and the control group were also found for feather mass and feather length of the P5s that had grown during the period of corticosterone application. With a median feather length of 165.5 mm (interquartile range [IQR]: 5.8) and a median feather mass of 310.2 mg (IQR: 19.5), P5s in the control group were both significantly longer (*P* < 0.001) and heavier (*P* < 0.001) than P5s in the CORT group (median feather length: 135.5 mm (IQR: 7.5); median feather mass: 172.7 mg [IQR: 20.8]). Furthermore, a delayed molting process was observed in pullets of the CORT group, where growth of the P5s was not completed until 17 wk of age. The P6s of the second feather generation were in different growth stages, whereas the P7 to P10 had not yet shed at this time (Figure 1A). In contrast, molting of the primaries was more advanced in the pullets of the control group, because only the P10 had not yet shed (Figure 1B). Similar effects of corticosterone applications or acute stress on feather growth and feather quality have been described in comparable studies in different bird species (Lattin et al., 2011; Romero and Fairhurst, 2016). However, the oral application of corticosterone did not induce the emergence of fault bars in primaries as shown in a previous study after psychological stress (Arrazola and Torrey, 2019). Thus, further investigations are needed to elucidate the role of HPA axis activity on feather development and fault bar formation.

**Figure 2:**
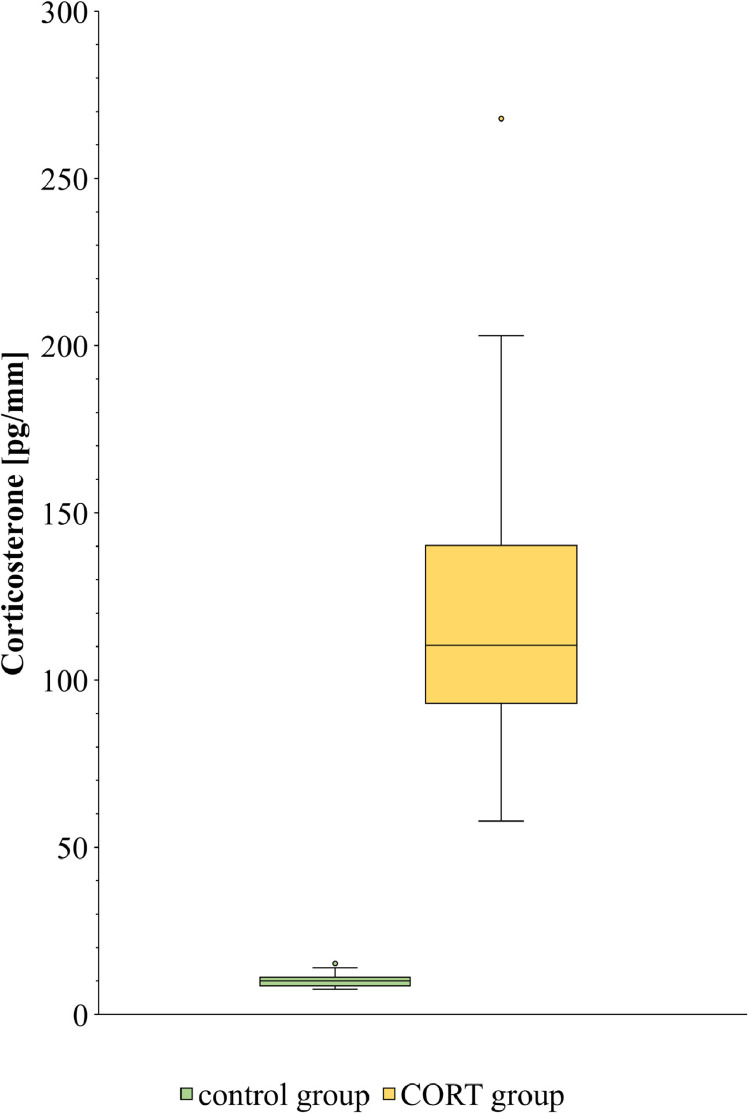
Corticosterone concentration in the fully grown P5 of LSL layer pullets after application of corticosterone (20 mg/l drinking water) during the growth phase of the respective feather. Median corticosterone concentration was 10.0 pg/mm in the control group (n = 20) and 110.3 pg/mm in the CORT group (n = 20).

The supplementation of drinking water with corticosterone increased fCORT in P5s of CORT pullets more than 10-fold compared with the control group (median: 110.3 pg/mm [IQR: 47.2] vs. 10.0 pg/mm [IQR: 2.5], *P* < 0.001, Figure 2). This resulted in a minimum-maximum range for fCORT of 7.52 to 15.24 pg/mm in the control group and 57.82 to 267.86 pg/mm in the CORT group, respectively. Thus, the highest fCORT concentration detected in the control group was 73.6 % lower than the lowest fCORT concentration detected in the CORT group.

This is the first study showing a long-term effect of corticosterone supplementation in drinking water on fCORT in layer pullets. The results demonstrate that experimentally increased systemic corticosterone concentrations over a period of seven weeks in layer pullets are reflected in corticosterone concentrations of feathers grown during that time. However, it should also be noted that the prolonged feather growth phase, the shorter feather length and lower feather mass of P5s in CORT pullets may contribute to increased fCORT because blood supply of the growing feather, and thus potential corticosterone incorporation, is maintained for a longer period of time.

The present results indicate that the measurement of fCORT may be a useful and minimally invasive tool for the assessment of long-term stress in chicken and provide the basis for further investigations on its use in animal welfare research. However, when fCORT is used as a stress indicator, sample preparation and detection methods need to be standardized to achieve comparable results (Häffelin et al., 2020). Further studies are required to investigate the influence of biotic and abiotic factors as well as husbandry conditions (e.g., stocking density, handling, environmental enrichment, lighting, parasite infestation, etc.) that may affect the incorporation and degradation or both of feather corticosterone before this method is applicable as a stress indicator in poultry farming.

## Appendix Supplementary materials

Image, application 1

## References

[bib0001] Arrazola A., Torrey S. (2019). The development of fault bars in domestic chicken (*Gallus gallus domesticus*) increases with acute stressors and individual propensity: implications for animal welfare. Anim. Welf..

[bib0002] Berk S.A., McGettrick J.R., Hansen W.K., Breuner C.W. (2016). Methodological considerations for measuring glucocorticoid metabolites in feathers. Conserv. Physiol..

[bib0003] Bortolotti G.R., Marchant T.A., Blas J., German T. (2008). Corticosterone in feathers is a long-term, integrated measure of avian stress physiology. Funct. Ecol..

[bib0004] Bortolotti G.R. (2010). Flaws and pitfalls in the chemical analysis of feathers: bad news-good news for avian chemoecology and toxicology. Ecol. Appl..

[bib0005] Carbajal A., Tallo-Parra O., Sabes-Alsina M., Mular I., Lopez-Bejar M. (2014). Feather corticosterone evaluated by ELISA in broilers: a potential tool to evaluate broiler welfare. Poult. Sci..

[bib0006] Häffelin K.E., Lindenwald R., Kaufmann F., Döhring S., Spindler B., Preisinger R., Rautenschlein S., Kemper N., Andersson R. (2020). Corticosterone in feathers of laying hens: an assay validation for evidence-based assessment of animal welfare. Poult. Sci..

[bib0007] Jenni-Eiermann S., Helfenstein F., Vallat A., Glauser G., Jenni L. (2015). Corticosterone: effects on feather quality and deposition into feathers. Methods Ecol. Evol..

[bib0008] Lattin C.R., Reed J.M., DesRochers D.W., Romero L.M. (2011). Elevated corticosterone in feathers correlates with corticosterone-induced decreased feather quality: a validation study. J. Avian Biol..

[bib0009] Leishman E.M., Freeman N.E., Newman A.E.M., van Staaveren N., Wood B.J., Harlander-Matauschek A., Baes C.F. (2020). Research note: quantifying corticosterone in turkey (*Meleagris gallopavo*) feathers using ELISA. Poult. Sci..

[bib0010] Monclús L., Tallo‑Parra O., Carbajal A., Quevedo M.A., Lopez‑Bejar M. (2020). Feather corticosterone in Northern Bald Ibis *Geronticus eremita*: a stable matrix over time able to predict reproductive success. J. Ornithol..

[bib0011] Reese L., Baumgartner K., von Fersen L., Merle R., Ladwig-Wiegard M., Will H., Haase G., Tallo-Parra O., Carbajal A., Lopez-Bejar M., Thöne-Reineke C. (2020). Feather corticosterone measurements of Greater Flamingos living under different forms of flight restraint. Animals.

[bib0012] Romero L.M., Fairhurst G.D. (2016). Measuring corticosterone in feathers: strengths, limitations, and suggestions for the future. Comp. Biochem. Physiol. Part A Mol. Integr. Physiol..

[bib0013] Scanes C.G. (2016). Biology of stress in poultry with emphasis on glucocorticoids and the heterophil to lymphocyte ratio. Poult. Sci..

[bib0014] Shini S., Kaiser P., Shini A., Bryden W.L. (2008). Differential alterations in ultrastructural morphology of chicken heterophils and lymphocytes induced by corticosterone and lipopolysaccharide. Vet. Immunol. Immunopathol..

[bib0015] Shini S., Kaiser P., Shini A., Bryden W.L. (2008). Biological response of chickens (*Gallus gallus domesticus*) induced by corticosterone and a bacterial endotoxin. Comp. Biochem. Physiol. B.

[bib0016] Shini S., Shini A., Huff G.R. (2009). Effects of chronic and repeated corticosterone administration in rearing chickens on physiology, the onset of lay and egg production of hens. Physiol. Behav..

[bib0017] Weimer S.L., Wideman R.F., Scanes C.G., Mauromoustakos A., Christensen K.D., Vizzier-Thaxton Y. (2018). An evaluation of methods for measuring stress in broiler chickens. Poult. Sci..

[bib0018] Wein Y., Bar Shira E., Friedman A. (2017). Avoiding handling-induced stress in poultry: use of uniform parameters to accurately determine physiological stress. Poult. Sci..

